# Evaluation of Efficacy, Safety, and Cognitive Profile of Amisulpride Per Se and Its Comparison with Olanzapine in Newly Diagnosed Schizophrenic Patients in an 8-Week, Double-Blind, Single-Centre, Prospective Clinical Trial

**DOI:** 10.5402/2012/703751

**Published:** 2012-03-14

**Authors:** Ganesh R. Pawar, P. Phadnis, A. Paliwal

**Affiliations:** ^1^Department of Pharmacology, MGM Medical College, Indore 452001, India; ^2^Department of Psychiatry, MY Hospital, Indore 452001, India

## Abstract

*Background*. Impaired cognitive functions in schizophrenia are the major deciding factors in response to treatment. Conventional antipsychotics have minimal impact on cognitive dysfunctions and are associated with adverse effects. Atypical antipsychotics have shown promise in treatment of cognitive and negative symptoms of schizophrenia. Efforts are underway to find out the best drug amongst atypical antipsychotics. *Objective*. To compare efficacy, safety, and cognitive profile of amisulpride and olanzapine in the treatment of acute psychotic exacerbations of schizophrenia. *Method*. A prospective, randomized, double-blind, single-center, 8-week clinical trial we used. *Subjects and Treatments*. Seventy four patients were treated for two months with either amisulpride (400–800 mg/d) or olanzapine (10–20 mg/d). *Statistics*. Mann Whitney *U* test we used for independent samples with *P* < 0.05 taken as significant. *Results*. Brief psychiatric rating scale (BPRS) was used as a primary measure of efficacy. Other measures of efficacy and safety were also evaluated. Both amisulpride and olanzapine groups showed equivalent improvement in psychotic symptoms on BPRS scale. Less than five percent of patients suffered adverse effects only to withdraw from the study. Olanzapine group showed statistically significant (*P* < 0.05) weight gain compared with amisulpride group. Amisulpride group showed significant improvement (*P* < 0.05) in various cognitive parameters as compared to olanzapine group.

## 1. Introduction

Most of the patients of schizophrenia have impaired cognitive function [1–3]. Cognitive impairment is associated with poor functional outcome and long-term prognosis [4, 5]. These impairments also determine quality of life and medical and social cost of schizophrenia [6, 7].

Systematic analyses have shown that conventional antipsychotics have a modest benefit in improving cognitive dysfunctions [8]. Also, current treatment of schizophrenia with these typical antipsychotics is limited by the side effects of the drugs. Side effects like Parkinsonian syndrome [9, 10] and tardive dyskinesia [11] occur in a significant number of patients only to deteriorate the quality of life.

With the arrival of newer atypical antipsychotics that are claimed to have low incidence of extrapyramidal effects [12] and provide a more significant improvement in cognitive dysfunctions than conventional drugs [13], The scenario of schizophrenia seems to change though results are not entirely consistent [14].

Though these atypical drugs share the property of blocking dopamine D_2_ receptors in limbic system, they have different overall pharmacological profile.

Amisulpride is an atypical antipsychotic with selective affinity for D_2_ and D_3_ receptors [15], with efficacy in positive and negative schizophrenic symptoms, and with low incidence of extrapyramidal symptoms [16]. This drug also shows improvement in neuropsychological performance in patients of schizophrenia [17, 18].

Olanzapine, on the other hand, acts on various monoamine receptors like 5-HT_2_ and D_2_ receptors [19]. It also shows low incidence of extrapyramidal side effects [20], efficacy in psychotic symptoms [16], and improvement in neuropsychological performance [21, 22].

We have performed a two-month, double-blind, randomized clinical trial of olanzapine versus amisulpride for treating acute exacerbation of schizophrenia. Overall objectives of study were to compare both treatments in terms of efficacy, safety, and impact on cognitive functions.

## 2. Methodology

The study was conducted in The Government Mental Hospital, Banganga, Indore, Madhya Pradesh, India. Inclusion started from May 2010 and ended on July 2011.

### 2.1. Patients

Patients of either sex in the age group of 18–45 years with a diagnosis of schizophrenia according to ICD 10 criteria were included. Both inpatients and outpatients were included in the study. At the screening and baseline visits, symptoms were assessed using brief psychiatric rating scale (BPRS scale) [23] and positive and negative syndrome scale (PANSS scale) [24]. A BPRS score of at least 36 and positive PANSS score more than negative was an essential criterion for randomization of patients.

Exclusion criteria were according to the labeling of the two drugs. Pregnant and lactating women were excluded and women of childbearing age were advised to use adequate means of contraception.

### 2.2. Treatment

Each patient was included in the study after applying inclusion and exclusion criteria and through clinical and psychiatric examination. Eligible patients were randomized to one of the treatment arms either amisulpride 400 mg/d or olanzapine 10 mg/d. Blinding was ensured by providing drugs in opaque envelopes with appropriate coding. The medicine doses were adjusted to 400–800 mg/d orally for amisulpride and olanzapine and 10–20 mg/d orally for olanzapine according to individual patient response and tolerability. Follow-up visits for efficacy, safety, and cognitive assessment were performed at 4 and 8 weeks.

Concomitant use of benzodiazepines, nonbenzodiazepine hypnotics (zolpidem, zopiclone), and anti-Parkinsonian drug, trihexyphenidyl, was allowed at the investigator's discretion.

### 2.3. Ethics

The study was conducted according to good clinical practices [European/ICMR guidelines]. Written informed consent was obtained from each patient. Institutional ethics committee approved the protocol. Patients were free to withdraw from the study at any time, due to any reason, and without effect on their treatment.

### 2.4. Outcome Parameters

Primary efficacy variable was the change from baseline in BPRS score. Other efficacy measure was PANSS score. Safety assessment included adverse event reporting and abnormal involuntary movement scale [25]. During each visit, a full clinical examination including recording of vital signs and body weight was done.

Cognitive assessment involved token test [26], Stroop test [27, 28], digit vigilance test [29], animal names test [29], and triads test [28].

### 2.5. Analysis

Subjects exposed to efficacy analysis were the intention-to-treat population, that is, all patients randomized and exposed to treatment and providing at least one postbaseline evaluation. Missing data due to premature discontinuation was handled using last-observation-carried-forward principle.

Analysis was performed using SPSS software version 14.

Mean change in BPRS and PANSS scale over the study period was compared in both the treatment arms using Mann Whitney *U* test for independent samples because of low sample size.

Mean change in scores of cognitive tests was compared in both the arms using Mann Whitney *U* test for independent samples. *P* < 0.05 was taken as significant.

## 3. Results

### 3.1. Study Population

Total of 81 patients were included (40 in amisulpride group and 41 in olanzapine group) with comparable baseline characteristics (Table 1). Out of these 74 patients provided at least one post treatment reading and these were the intention to treat population. 32 patients in amisulpride (80%) as well as olanzapine (78.04%) group completed the two months treatment. Principle reason for discontinuation was lack of efficacy (Table 2). Incidence of premature discontinuation was not different in both the groups.

### 3.2. Efficacy

During the study period change observed in BPRS score in amisulpride group was 16.80 (SD: 3.61) and in olanzapine group was 15.30 (SD: 2.69) (Table 3). This improvement was similar in both groups and was not statistically significant (*P* = 0.38).

Symptomatic improvement was also observed on PANSS scale in both the groups (*P* = 0.22) (Table 4). The improvement was mainly in positive symptoms.

### 3.3. Safety

Only one patient in amisulpride group suffered from adverse event to warrant his removal from the study.

There was no difference in score between baseline and end of two months in abnormal voluntary movement scale.

There was a more statistically significant (*P* < 0.001) weight gain observed in olanzapine group (2.09 kg) than in amisulpride group (0.94 kg).

### 3.4. Cognitive Assessment

#### 3.4.1. Token Test

The token test is a measure of verbal comprehension. It involves tokens of different color, size, and shapes. The test involves the capacity to follow spoken commands of varying complexity.

Amisulpride group performed significantly better (*P* = 0.02) than olanzapine group in this test (Table 5).

#### 3.4.2. Stroop Test: NIMHANS Version

This test is a measure of response inhibition. This test cannot be used in illiterate patients.

In this test there was statistically significant difference found between these two groups (*P* = 0.03) (Table 6).

#### 3.4.3. Triad Test

This test is for divided attention. It combines a verbal triads task with a tactual number identification task. Two tasks differ with reference to stimulus modality and nature of stimulus processing. Nature of response is similar in that both the tasks require verbal response.

Amisulpride group performed better than olanzapine group with a *P* value of 0.02 (Table 7). This test cannot be performed in illiterate subjects.

#### 3.4.4. Digit Vigilance Test

It is a test of attention, alertness, and mental processing capacity using a rapid visual tracking task.

Amisulpride group performed better in both aspects of test, that is, time factor (*P* < 0.001) and number of errors (*P* < 0.001) than olanzapine group (Table 8).

#### 3.4.5. Animal Names Test

This test is for category fluency, a form of verbal fluency.

Amisulpride group showed statistically significant (*P* < 0.001) improvement in this test than olanzapine group (Table 9).

## 4. Discussion

A major reason behind performing this study was to gather data on schizophrenic patients in India. There are very few studies performed in this regard especially covering the cognitive aspect. Cognitive tests used were designed by NIMHANS Institute, Banglore, especially for Indian population, which helped us to perform this study.

The study showed that amisulpride and olanzapine were equally effective in the treatment of acute exacerbation of schizophrenia as shown by BPRS scores. Both drugs showed comparable improvement over the study period of two months. As far as PANSS scale is concerned, improvement was mainly observed in positive symptomatology of schizophrenia. However, negative symptoms score was low at baseline and did not improve markedly during study in both the groups. The general psychopathology symptom subscale in PANSS also showed improvement during the study.

In case of safety, major parameters analyzed were weight gain and extrapyramidal symptoms. Weight gain is typically regarded as common side effect of antipsychotic drugs [30]. This is particularly important with newer antipsychotic drugs [31–33]. Olanzapine group showed a significant weight gain (mean of 2.09 kgs) as compared to amisulpride group (mean of 0.9 kgs). Excessive weight gain with olanzapine is a well-characterized side effect of this drug [34, 35] and may increase the subsequent risk of cardiovascular disease, diabetes, and hyperlipidemia [36]. This may be a deciding factor in patient compliance in the long term. Difference in weight gain in both the drugs may be explained by the mechanisms of action of both. Weight gain is claimed to involve serotonergic mechanism, and olanzapine is a potent antagonist at 5-HT_2_ receptors [37]. Amisulpride being a very specific dopaminergic antagonist may not share the weight gain property. With respect to extrapyramidal side effects, our study did not show emergence of these in both treatment groups. This is consistent with previous studies of these two agents [34–37].

Amisulpride group fared better in cognitive aspects of study, that is, divided attention, response inhibition, verbal comprehension, and verbal fluency. Improvement observed in olanzapine group was modest as compared to amisulpride group. Previous studies [13, 17, 38] have shown that agents like olanzapine, which have affinity for 5-HT_2_, lack effect on learning and memory performance in patients of schizophrenia. While our study shows results consistent with these studies, our results are in disagreement with a large Canadian open-label study [39], which showed improvement with olanzapine, which may be due to low sample size in our study. Considering the differences between these two agents in terms of effect on cognition, the following explanation may clear the difference.

Dopamine projections to the prefrontal cortex comprising the mesocortical dopamine system are essential for normal cognition.

Amisulpride is a benzamide with high affinity for dopamine D_2_, and D_3_, receptors without affinity for serotonin, muscarinic, or alpha-adrenergic receptors.

At low doses (100–300 mg/d), amisulpride binds preferentially on D_2_/D_3 _presynaptic autoreceptors, increasing dopaminergic transmission in the prefrontal cortex, which is believed to be associated with improvement of cognitive symptoms [40]. Selective binding of amisulpride also makes it free of side effects like sedation and weight gain seen with blockade of other receptors.

It was proved in a previous study [38, 41] that serotonin antagonism as in olanzapine was not necessary for improvement in cognitive dysfunctions and was responsible for some of the side effects like weight gain seen with olanzapine. Olanzapine improves verbal learning and memory, verbal fluency, and executive function, but not attention, working memory, or visual learning and memory.

## 5. Conclusion

To conclude, amisulpride and olanzapine show equivalent efficacy in improving psychotic symptoms of schizophrenia. Amisulpride gains an upper hand in preserving body weight and in improvement of cognitive functions.

## Figures and Tables

**Table 1:** Baseline characteristics of patients exposed to drugs (categorical variables presented as absolute patient numbers and quantitative variables presented as mean [SD] values).

	Amisulpride	Olanzapine
Age [years]		
Mean [SD]	29.1 [7.05]	31.4 [8.16]
Gender		
[Male/female]	18/14	17/15
Weight [kg]	52.5 [8.78]	49.7 [9.32]
Duration of illness [weeks]	11.3 [3.22]	10.8 [3.54]
Mean BPRS score	54.7 [5.92]	57.5 [7.73]

**Table 2:** Patient flow through study.

	Amisulpride	Olanzapine
Patients randomized	40	41
Completed	32 [80%]	32 [78.04%]
Premature withdrawal:	8 [20%]	9 [21.96%]
Lost to followup	1	1
Lack of efficacy	3	4
Adverse event	1	0
Unco-operative	1	2
Others	2	1

**Table 3:** BPRS score throughout the study (scores presented as mean [SD] values).

Visit	Amisulpride group	Olanzapine group
1	54.7 [5.92]	57.5 [7.73]
2	47.1 [5.72]	49.3 [7.20]
3	37.9 [6.81]	42.2 [7.74]
Mean change from baseline	16.8 [3.61]	15.3 [2.69]

**Table 4:** Mean change in PANSS score during study period (scores presented as mean [SD] values).

	Amisulpride	Olanzapine
PANSS scale		
Total score	−16.34 [3.31]	−18.31 [5.71]
Positive score	−6.47 [1.68]	−7.89 [2.67]
Negative score	−2.25 [0.880]	−2.61 [1.14]
General psychopathology	− 7.72 [1.91]	−7.71 [2.89]

**Table 5:** Scores in each visit for token test (scores presented as mean of the scores in the test [SD] values).

Token test	Amisulpride	Olanzapine
Visit 1	14.18 [3.37]	14.34 [3.64]
Visit 2	18.81 [3.22]	17.78 [3.37]
Visit 3	22.40 [3.21]	21.12 [3.45]
Mean change	8.26 [1.56]	6.78 [2.09]

**Table 6:** Scores in each visit for Stroop test (scores presented as mean of the difference between time taken to read colors minus time taken to read words [SD] values).

Stroop test	Amisulpride	Olanzapine
Visit 1	288.16 [64.78]	332.47 [91.89]
Visit 2	238.50 [58.98]	290.72 [84.73]
Visit 3	205.19 [60.21]	257.38 [83.81]
Mean change	83.56 [14.50]	75.09 [18.07]

**Table 7:** Scores in visit for triads test (scores presented as mean number of errors [SD] values).

Triads test	Amisulpride	Olanzapine
Visit 1	20.47 [2.52]	19.84 [3.71]
Visit 2	17.38 [2.55]	17.03 [3.72]
Visit 3	14.97 [2.59]	15.53 [4.04]
Mean change	5.50 [1.45]	4.31 [1.25]

**Table 8:** Scores in each visit for digit vigilance test (scores presented as mean time taken to complete the test and mean number of errors performed [SD] values).

Digit vigilance test	Amisulpride	Olanzapine
Time [in seconds]		
Visit 1	684.53 [69.90]	613.06 [66.79]
Visit 2	639.78 [69.86]	577.31 [63.43]
Visit 3	607.50 [69.57]	554.16 [63.24]
Mean change	77.03 [15.97]	58.72 [14.91]

Errors		
Visit 1	217.94 [43.42]	234.19 [44.6]
Visit 2	196.69 [42.74]	218.69 [43.25]
Visit 3	183.03 [42.14]	206.59 [43.62]
Mean change	35.87 [8.35]	27.91 [7.78]

**Table 9:** Scores in each visit for animal names test (scores presented as mean number of new words generated [SD] values).

Animal names test	Amisulpride	Olanzapine
Visit 1	9.06 [1.91]	8.94 [1.99]
Visit 2	13.44 [2.06]	11.81 [2.26]
Visit 3	15.88 [1.71]	13.72 [2.18]
Mean change	6.81 [1.57]	4.78 [1.64]
